# Crimean-Congo Hemorrhagic Fever Virus Circulation in Wild European Rabbits, Portugal, 2018−2023

**DOI:** 10.3201/eid3110.250184

**Published:** 2025-10

**Authors:** Carolina Baptista, Nuno Santos, Laurence Vial, Ferran Jori

**Affiliations:** University of Porto Research Centre in Biodiversity and Genetic Resources, Vairão, Portugal (C. Baptista); CIBIO (Research Centre in Biodiversity and Genetic Resources)-inBIO Associate Laboratory Research Network, Vairão (N. Santos); UMR ASTRE, CIRAD (French Agricultural Research Centre for International Development), Montpellier, France (L. Vial, F. Jori)

**Keywords:** Crimean-Congo hemorrhagic fever virus, viruses, European rabbits, *Oryctolagus cuniculus*, Portugal

## Abstract

Crimean-Congo hemorrhagic fever virus is considered a public health risk in southwestern Europe. We surveyed serum samples from 667 European rabbits across Portugal, a rabbit species known to host immature *Hyalomma lusitanicum* ticks. We found low levels of virus antibodies (>1%), with a localized cluster reaching 5.77% in southern populations.

Crimean-Congo hemorrhagic fever virus (CCHFV) is a highly pathogenic tickborne pathogen able to cause severe hemorrhagic fever that has a high case-fatality rate in humans (*1*). Since the virus’ first detection in southwestern Europe in 2010 (*2*), CCHFV has emerged as a formidable public health risk. Reports from Spain have identified *Hyalomma lusitanicum* ticks as reservoirs and vectors of CCHFV (*2*–*4*) and have suggested circulation and maintenance of the virus at local levels to be related to animal abundance (*5*). Mammals infected by tick bites become viremic for 2–10 days and develop a persistent immune response (*4*), making serologic surveys an effective tool for monitoring CCHFV dynamics (*2*,*4*–*6*).

Wild lagomorphs, and particularly European rabbits (*Oryctolagus cuniculus*), are key hosts of immature stages of *H*. *lusitanicum* ticks (*7*) and are expected to play a critical role in CCHFV epidemiology (*8*). However, prior reports have not established clear evidence of natural exposure of lagomorphs to CCHFV in Europe (*8*). Our study aimed to fill this gap through a serologic survey of rabbit populations from Portugal.

During May 2018−December 2023, we sampled 667 wild rabbits across 20 sites throughout mainland Portugal (average 33.4 ± 46.1 [standard deviation] rabbits per site) (Figure). We selected 8 longitudinal sites on the basis of their high rabbit abundance. Twelve cross-sectional sites included hunted rabbits from ongoing studies. Study sites consisted of mixed agro-forestry landscapes with variable rabbit and wild ungulate abundance. Samples (1 per animal) encompassed blood from live-captured rabbits in a longitudinal capture-recapture study (n = 472) and hunted rabbits (n = 195), including 71 dried blood spots (DBS). We collected blood samples by way of the saphenous vein (live rabbits) or the thoracic cavity (hunted rabbits) and centrifuged samples at 2,000 × *g* for 10 minutes to obtain serum. We collected blood from the thoracic or abdominal cavities as DBS in Whatman Protein Saver 903 cards (Cytiva, https://www.cytivalifesciences.com), dried at room temperature (2–6 weeks) before being shipped to the laboratory. We stored serum samples and DBS at −20°C prior to processing them for CCHFV detection using a commercial ELISA with recombinant purified CCHFV nucleoprotein (IDScreen, Double Antigen Multi species; https://www.innovative-diagnostics.com), following the manufacturer’s instructions.

**Figure:**
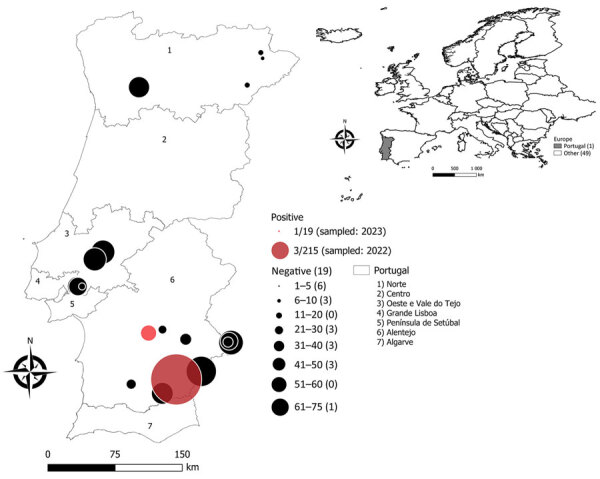
Study sites in Portugal, sample sizes, and locations of seropositive rabbits in study of Crimean-Congo hemorrhagic fever virus circulation in wild European rabbits, Portugal, 2018−2023. Inset map shows location of Portugal in Europe (gray shading).

We performed a univariate analysis because of a low number of positive ELISA results. We evaluated associations between seropositivity and individual categorical variables (year, month, sex, and age class) using Fisher exact test. We considered a p value <0.05 to be significant. We conducted statistical analyses in R v4.3.1 (The R Project for Statistical Computing, https://www.r-project.org) and QGIS v3.38.2 (https://qgis.org) for mapping the results.

We detected 4 animals with CCHFV antibodies (n = 667), resulting in an overall prevalence of 0.60% (95% CI 0.23%–1.53%). We collected the 4 positive samples from 2 sites located 74 km apart in southern Portugal (Figure), within an area previously identified as highly active for the circulation of CCHFV in wildlife (*5*). Those samples encompassed a DBS from a rabbit in 2023 (data not available) and 3 serum samples from rabbits captured alive in 2022 (1 female and 1 male in June, 1 female in July, all juveniles). None of the seropositive rabbits were later recaptured. In this location, longitudinal monitoring of the same population identified a sudden increase of seroprevalence between 2 consecutive years, ranging from 0 (0/86 rabbits) in 2021 to 5.77% (95% CI 1.98–15.64%; 3/52 rabbits;) in 2022 (Table), before dropping again to undetectable levels in 2023 (0/78 rabbits). We analyzed 143 rabbits in 9 other sites from Portugal collected in 2022 without detecting any seropositivity, suggesting that CCHFV circulation was very localized. Given the rabbit density data obtained by capture-recapture methods in that same population (peak densities of 3.0–3.8 rabbits/hectare in 2021–2023) (*9*), we estimated the captured rabbits to represent 40%–75% of the total population yearly. Therefore, we inferred that the probability of CCHFV being undetected in 2021 and 2023 ranged from 1% to 10%. (https://epitools.ausvet.com.au/freedomss). Overall, our data suggest that CCHFV circulation in rabbit populations in Portugal was highly localized in space and time, with a sudden increase in a specific southern location (Mértola) in 2022.

**Table:** Univariate analysis of binomial serologic results in study of Crimean-Congo hemorrhagic fever virus circulation in wild European rabbits, Portugal

Category	Seropositive, no.	Sample size, no.	Prevalence, % (95% CI)	p value*
Sex				Referent
** M**	1	260	0.37 (0.07–2.15)	
** F**	2	289	0.71 (0.21–2.76)	
** Unknown**	1	122	0.82 (0.14–4.50)	
Age				**0.041**
** Adult**	0	326	0 (0–1.16)	
** Subadult**	0	104	0 (0–3.56)	
** Juvenile**	3	127	2.36 (0.81–6.72)	
** Unknown**	1	110	0.90 (0.16–4.97)	
Year				0.743
** 2018**	0	60	0 (0–6.02)	
** 2019**	0	51	0 (0–7.00)	
** 2020**	0	59	0 (0–6.11)	
** 2021**	0	127	0 (0–2.94)	
** 2022**	3	195	1.54 (0.52–4.42)	
** 2023**	1	175	0.57 (0.10–3.17)	
Month of sample collection				0.519
** Jan–Apr**	0	45	0 (0–7.87)	
** May–Aug**	3	299	1.00 (0.34–2.91)	
** Sep–Dec**	1	314	0.31 (0.06–1.78)	
** Unknown**	0	9	0 (0–29.91)	

*By Fisher exact test. Bold indicates significance (p<0.05).

Our results highlight the need for further studies to understand the ecologic and epidemiologic role of wild rabbits in the dynamics of *Hyalomma* tick populations and CCHFV circulation. Considering the preference of immature *H. lusitanicum* ticks for lagomorphs in the Iberian Peninsula, we anticipated the detected exposure of rabbits to CCHFV (*7*). Nevertheless, previous surveys in wild rabbit populations from areas of suspected active circulation in Spain (*5*) did not detect any evidence of CCHFV exposure. Given our results of 10% between-cluster prevalence and 5% within-cluster seroprevalence, we estimated that aiming for 95% cluster sensitivity, a minimum sample of 31 rabbits per cluster would be required to detect exposure to CCHFV in areas of active viral circulation (https://epitools.ausvet.com.au/twostagefreedomsstwo). Therefore, when planning future surveys in wild rabbits, we recommend implementing a 2-stage cluster sampling approach (*10*) to better detect spatiotemporally clustered CCHFV antibodies. 
